# Adulteration of Food and Drugs

**Published:** 1860-05

**Authors:** 


*Adulteration of Food and Drugs*—that most dastardly and nefarious description of commercial roguery which impunity and long practice have combined to bring to such perfection in England—is fraught with difficulty and visited with heavy penalties in Paris. The vigilance of the Paris police almost suffices to ensure the public against the evils of slow poisoning which *we* are compelled to undergo to an extent which may account for many of the diseases prevalent in this country, and generally attributed to climate. Surely no supervision can be deemed superfluous, and no chastisment too severe, which may tend to obviate such horrors as we know to be practised on the English public, especially on the poor. When a Paris shopkeeper is caught either diluting, adulterating, or fraudulently mixing his merchandise, or in selling short weight or measure, he is first heavily fined, and then condemned to a public confession of his guilt by stating the particulars on a large placard, conspicuously exhibited in his shop during the pleasure of the court; so that he himself informs his customers he has been practicing on their credulity, tampering with their health, and picking their pockets. —*Realities of Paris Life.*

